# Posterior transcallosal intervenous-interforniceal approach to a periaqueductal tumor

**DOI:** 10.3171/2019.10.FocusVid.19457

**Published:** 2019-10-01

**Authors:** David S. Hersh, Katherine N. Sanford, Frederick A. Boop

**Affiliations:** 1 Department of Neurosurgery, University of Tennessee Health Science Center;; 2 Le Bonheur Children’s Hospital; and; 3 Semmes Murphey Clinic, Memphis, Tennessee

**Keywords:** brainstem, aqueductal, transcallosal, fornix, video

## Abstract

Described by Dandy in 1921, the posterior interhemispheric transcallosal approach provides an operative corridor to the pineal region, posterior third ventricle, and upper midbrain. Intervenous-interforniceal and paravenous-interforniceal variants have been utilized for midline and paramidline pathology, respectively. The intervenous-interforniceal variant capitalizes on the natural separation of the internal cerebral veins, which are found medial to the forniceal crura at this level, to provide a safe corridor to the tumor while minimizing the risk of injury to the fornices. Here, the authors describe a posterior interhemispheric transcallosal approach using the intervenous-interforniceal variant for resection of a periaqueductal pilocytic astrocytoma.

The video can be found here: https://youtu.be/mtQKEXEveTg.

## Transcript

In this video, we describe a posterior, interhemispheric, transcallosal intervenous-interforniceal approach to a periaqueductal tegmental tumor.

### 0:33 History

The patient was a 15-year-old female who presented with headaches. Imaging revealed obstructive hydrocephalus secondary to a mass centered in the periaqueductal region of the tegmentum.

### 0:57 Preop imaging 1

T1- and T2-weighted MRI sequences demonstrated a T2 hyperintense mass centered along the periaqueductal region, measuring approximately 1.3 cm in all three planes. There was nodular rim enhancement of the lesion. Mild tumoral edema was visualized extending along the posterior and medial margins of the mass, with mass effect resulting in obstruction of the cerebral aqueduct.

### 1:22 Preop imaging 2

Coronal views demonstrated that the posterior commissure was draped over the mass, and sagittal views indicated that the tectum was displaced posteriorly by the lesion.

### 1:36 Preoperative course

Initially, an endoscopic third ventriculostomy was performed. Using the same burr hole, an endoscopic biopsy of the mass was attempted. However, an ependymal layer overlying the mass resulted in poor visualization and localization of the lesion, and a biopsy could not be performed safely. Therefore, an open biopsy and resection of the mass via a posterior transcallosal intervenous-interforniceal approach was recommended. More posteriorly directed approaches, such as the supracerebellar infratentorial approach and the occipital transtentorial approach, were considered suboptimal due to the posterior displacement of the tectum by the tumor, placing this structure in the path of surgical dissection.

### 2:34 Relevant anatomy

A key feature of the posterior transcallosal intervenous-interforniceal approach is the anatomical relationship of the internal cerebral veins and forniceal crura. There is a natural separation of the internal cerebral veins at the posterior aspect of the corpus callosum, just proximal to their confluence with the vein of Galen. The forniceal crura are always lateral to the internal cerebral veins at this level. Therefore, by maintaining a plane of dissection between the two internal cerebral veins, the fornices remain protected.

### 3:16 Coronal view

This anatomical relationship is highlighted by the patient’s preoperative imaging. The coronal T2-weighted MRI demonstrates the separation of the forniceal crura posterior to the body of the fornix, and the lateral location of the crura relative to the internal cerebral veins. This view illustrates that a midline, posterior interhemispheric, transcallosal intervenous-interforniceal approach would provide a direct path to the underlying tumor.

### 3:51 Positioning and opening

With this in mind, a posterior transcallosal intervenous-interforniceal approach was performed. The patient was placed in the MRI-compatible carbon graphite pin head holder. She was secured in the supine position with her neck flexed. Using stereotactic navigation, the sagittal sinus was marked and a modified zigzag bifrontal incision was marked at the frontoparietal junction. Burr holes were placed on either side of the midline, and a bifrontal craniotomy was turned at the posterior aspect of the frontal region. The dura was opened on the right in a C-shaped fashion and flapped towards the sagittal sinus.

### 4:37 Initial exposure

Upon opening the dura, the brain was noted to be quite full. The frameless stereotactic navigation system was used to make a small corticotomy in the parietal region and a catheter was guided into the right lateral ventricle. Using the frameless stereotactic system, we opened the posterior corpus callosum using bipolar coagulation. Once the callosotomy was complete, we entered the body of the right lateral ventricle.

### 5:13 Septostomy

The choroid plexus and fornix were identified. Next, the septum pellucidum was fenestrated and opened. This allowed us to view both fornices as they separated with the internal cerebral veins at the posterior aspect of the corpus callosum.

### 5:44 Opening the velum interpositum and tela choroidea

After confirming our location with our stereotactic neuronavigation, we opened into the vellum interpositum. The velum interpositum forms the roof of the third ventricle and contains the paired internal cerebral veins and medial posterior choroidal arteries. We continued to expose the internal cerebral veins. The thin, translucent tela separating the internal cerebral veins came into view and was opened. This facilitated separation of the internal cerebral veins.

### 7:27 Tumor biopsy and resection

Once the roof of the third ventricle had been fully opened, the white matter of the posterior commissure was transected. Gray, gelatinous tumor was immediately encountered. Biopsies were taken for permanent specimen. The ultrasonic aspirator was then brought into the field and used to gently remove the tumor by suction. We continued to slowly resect tumor using the ultrasonic aspirator. Eventually, we were able to open the cerebral aqueduct. However, at its margins, the tumor appeared to become infiltrative and appeared to involve the periaqueductal region. This area felt firm. Additionally, it did not aspirate easily with the ultrasonic aspirator. Given that this region appeared to be infiltrated on imaging and was only visualized on the T2 sequences, we did not pursue further resection in this area. Hemostasis was secured, the wound was irrigated, and all cottonoids and Gelfoam were removed.

### 9:51 Intraoperative MRI

Intraoperative MRI revealed a gross-total resection of the enhancing portion of the tumor, though there was a significant residual infiltrative component in the periaqueductal region. Given that the tumor appeared to be infiltrative at this level, the decision was made not to resect further.

### 10:14 Postoperative exam

The patient was noted to have intact extraocular movements upon awakening from surgery. She initially reported mild diplopia, which resolved by discharge on postoperative day 3.

### 10:27 Histology

The histology was consistent with a pilocytic astrocytoma, and discussions regarding adjuvant treatment are currently pending.
